# Unbiased Characterization of *Anopheles* Mosquito Blood Meals by Targeted High-Throughput Sequencing

**DOI:** 10.1371/journal.pntd.0004512

**Published:** 2016-03-10

**Authors:** Kyle Logue, John Bosco Keven, Matthew V. Cannon, Lisa Reimer, Peter Siba, Edward D. Walker, Peter A. Zimmerman, David Serre

**Affiliations:** 1 Genomic Medicine Institute, Cleveland Clinic, Cleveland, Ohio, United States of America; 2 Department of Biology, Case Western Reserve University, Cleveland, Ohio, United States of America; 3 Center for Global Health and Diseases, Case Western Reserve University, Cleveland, Ohio, United States of America; 4 Department of Microbiology and Molecular Genetics, Michigan State University, East Lansing, Michigan, United States of America; 5 Papua New Guinea Institute of Medical Research, Madang, Papua New Guinea; 6 Liverpool School of Tropical Medicine and Hygiene, Liverpool, United Kingdom; 7 Papua New Guinea Institute of Medical Research, Goroka, Papua New Guinea; Johns Hopkins University, UNITED STATES

## Abstract

Understanding mosquito host choice is important for assessing vector competence or identifying disease reservoirs. Unfortunately, the availability of an unbiased method for comprehensively evaluating the composition of insect blood meals is very limited, as most current molecular assays only test for the presence of a few pre-selected species. These approaches also have limited ability to identify the presence of multiple mammalian hosts in a single blood meal. Here, we describe a novel high-throughput sequencing method that enables analysis of 96 mosquitoes simultaneously and provides a comprehensive and quantitative perspective on the composition of each blood meal. We validated *in silico* that universal primers targeting the mammalian mitochondrial 16S ribosomal RNA genes (16S rRNA) should amplify more than 95% of the mammalian 16S rRNA sequences present in the NCBI nucleotide database. We applied this method to 442 female *Anopheles punctulatus s*. *l*. mosquitoes collected in Papua New Guinea (PNG). While human (52.9%), dog (15.8%) and pig (29.2%) were the most common hosts identified in our study, we also detected DNA from mice, one marsupial species and two bat species. Our analyses also revealed that 16.3% of the mosquitoes fed on more than one host. Analysis of the human mitochondrial hypervariable region I in 102 human blood meals showed that 5 (4.9%) of the mosquitoes unambiguously fed on more than one person. Overall, analysis of PNG mosquitoes illustrates the potential of this approach to identify unsuspected hosts and characterize mixed blood meals, and shows how this approach can be adapted to evaluate inter-individual variations among human blood meals. Furthermore, this approach can be applied to any disease-transmitting arthropod and can be easily customized to investigate non-mammalian host sources.

## Author Summary

Female mosquitoes require a blood meal to acquire the nutrients necessary for egg production. While feeding on host species, mosquitoes can transmit pathogens that cause several diseases including malaria, lymphatic filariasis and dengue. Understanding the mosquito host choice is important to better implement control strategies to reduce mosquito populations and therefore transmission of disease. Currently, the majority of methods for evaluating host species only test for the presence of pre-selected, expected hosts. Here, we describe an unbiased assay that combines amplification of any mammalian DNA with high-throughput sequencing to comprehensively characterize the composition of mosquito blood meals. We applied this approach to *Anopheles* mosquitoes collected in Papua New Guinea and observed that they fed on expected (humans, dogs and pigs) and unexpected hosts (mice, bats, marsupials). In addition, we show that 16.3% of mosquitoes fed on multiple hosts, from the same or different species. Overall, this approach enables unbiased characterization of mosquito blood meals and can be easily applied to significantly improve our understanding of the feeding behavior of any disease-transmitting insect.

## Introduction

Many insects require a blood meal to complete their gonotrophic cycle. By feeding successively on different hosts, these insects can transmit blood borne pathogens that cause diseases responsible for significant burden on global health [1, 2]. In particular, insects that seek human blood meals are vectors of devastating diseases such as malaria, dengue fever, sleeping sickness, filariasis, leishmaniasis, typhus and plague. Understanding the complex blood feeding patterns of the insects transmitting these human diseases is crucial for developing and prioritizing vector-based control program activities and identifying potential unrecognized disease reservoirs, and thus for reducing disease transmission and burden.

The blood meals of arthropods have traditionally been analyzed using serological techniques such as ELISA or precipitin tests [3–5]. While these methods have provided valuable information, they have limited taxonomic resolution as they are generally only able to characterize hosts at the order or family levels [6]. In addition, since these approaches test for the presence of a protein from a specific organism, they only test for absence/presence of organisms that are *a priori* believed to be blood meal hosts. More recently, a number of PCR-based molecular techniques have been developed to characterize host blood meals ([7] and references within) and determine the blood feeding preference of mosquitoes [8–11], ticks [12–14], sandflies [15–17] and Tsetse flies [18, 19]. While these PCR-based approaches enable rigorous identification of the host species, they typically focus on species-specific amplification of putative hosts and therefore are not designed to identify novel, unanticipated host blood sources. In addition, the detection of mixed blood meals (i.e., when an insect feeds on more than one host) by these approaches is complicated as the dominant host signal can completely overwhelm signals from other minor hosts. These limitations may have biased our understanding of the transmission of many vector-borne diseases and have prevented identification of important disease reservoirs.

Beyond the identification of the host species, it may also be important to understand which individuals of a given species are being fed upon: for example, knowing whether an insect preferentially bites specific individuals or, in contrast, feeds on multiple individuals per night, could influence our assessment of disease transmission. A number of studies have used microsatellites or other polymorphic genetic markers to generate individual DNA fingerprints from human blood meals of mosquitoes [20–25] and lice [26, 27]. However, interpretation of these data can become complicated if DNA from more than one individual is present in a single blood meal.

*Anopheles punctulatus sensu latu* (*s*.*l*) mosquitoes are the principal vectors of malaria and lymphatic filariasis in Papua New Guinea (PNG) and the South Pacific [28]. There are 13 sibling species in *An*. *punctulatus s*.*l*, five of which are major disease vectors: *An*. *punctulatus s*.*s*., *An*. *koliensis*, *An*. *farauti s*.*s*., *An*. *hinesorum* and *An*. *farauti* 4. While these species have been little studied, they are generally characterized as unspecialized with regards to their feeding behaviors and ecological preferences [29] and shown to feed roughly indiscriminately on humans, dogs and pigs that are the most abundant species found in PNG villages [30, 31].

Here, we describe a novel approach using next-generation sequencing technology to analyze the blood meal composition of individual mosquitoes in an unbiased manner. We first amplify DNA extracted from a single female mosquito using universal primers targeting the mammalian mitochondrial (mt) 16S rRNA genes. Following individual barcoding, PCR products from up to 96 mosquitoes are pooled and simultaneously sequenced using Illumina high-throughput sequencing methods. We also use the same approach to interrogate whether individual mosquitos fed on more than one person by sequencing a highly polymorphic region of the human mt hypervariable region I. We applied this approach to 442 *Anopheles punctulatus sensu lato* (*s*.*l*) mosquitoes captured in five villages of the Madang Province of Papua New Guinea and provide evidence that (i) *Anopheles punctulatus s*.*l*. mosquitoes feed on a variety of mammalian species, including several unanticipated hosts, and (ii) *Anopheles punctulatus s*.*l*. mosquitoes frequently feed on multiple mammalian hosts. We also show how this assay can be easily customized to examine the number of individual hosts within a specific species. Overall, our results illustrate the potential of this approach to comprehensively characterize host species for any blood feeding arthropods, to identify reservoirs of pathogens and to provide opportunities for developing better evidence-based strategies to decrease transmission of important infectious diseases.

## Methods

### Ethics

This study was approved by the Papua New Guinea Institute of Medical Research Institutional Review Board (1203) and PNG Medical Research Advisory Board (12.05).

### Sample collections

We collected mosquitoes from the villages of Dimer, Wasab, Kokofine, Mirap and Matukar in the Madang province of Papua New Guinea (PNG) in June and August 2012. In each village, field technicians collected mosquitoes between 1800 and 0600 using barrier screens as described by Burkot *et al* [32]. These screens were manually searched every 20 minutes and resting mosquitoes were captured from the screens using an aspiration device. After collection, the sex and species of each mosquito were determined by morphology as previously described [33]. All male mosquitoes and non-*Anopheles* mosquitoes were discarded. We visually classified each female *Anopheles* mosquito as non-fed, partially-fed or fully-fed by examining the size and coloration of their abdomen. We individually stored each mosquito in an Eppendorf tube containing silica gel as desiccant.

### DNA isolation and molecular species identification

We extracted DNA from individual mosquitoes using a 96 well Qiagen DNeasy blood and tissue kit as previously described [34]. Mosquito species identification was determined using a PCR-based assay that evaluates species-specific polymorphisms in the ribosomal RNA internal transcribed spacer unit 2 (ITS2) [35].

### *In silico* assessment of mammalian mt 16S rRNA primers

To test the range of mammals that should be amplified using mt 16S rRNA primers [36], we conducted an *in silico* analysis using the primerTree package. We also conducted *in silico* analyses for two other previously published primers, cytochrome oxidase I (COI) and cytochrome b (Cytb) that have been previously used for mosquito blood meal identification [37]. Since the 16S rRNA locus appeared to be the most informative for our purposes (S1 Fig), we restricted our further analyses to this locus. Briefly, we performed primer-BLAST searches using the mammalian mt 16S rRNA primer sequences against the NCBI nucleotide database using default parameters but allowing for up to three mismatches in the primer sequences. In our search, we set the maximum number of blast hits retrieved to 10,000 and retrieved the taxonomical information of each sequence retrieved. As this search can be biased by recent release of many DNA sequences from a specific taxon, we performed this search separately for each mammalian order. We then calculated how many different species were obtained from each order to calculate the total number of mammalian species likely to be amplified by this primer pair. Note that, when conducting the search without any taxonomic restrictions, these mammalian primers were also predicted to amplify amphibian and fish 16S rRNA genes.

To estimate the total number of mammalian species for which the targeted locus has been sequenced and deposited in NCBI, we randomly selected one DNA sequence from each mammalian family and used BLAST searches to identify similar DNA sequences in the NCBI nucleotide database (accessed on July 2015). We filtered out any DNA sequence from the database that did not contain the primer sequences (allowing for up to three mismatches). We then merged the results from the searches performed in each family and counted how many unique species were observed. These analyses provided us with the total number of mammalian species that should be amplified if the primers were truly universal.

We also evaluated whether the 16S rRNA primers amplified sufficiently informative DNA sequences to support rigorous species identification (i.e., whether related species could be distinguished). First, we retrieved the mammalian DNA sequence alignment from the primerTree analysis and calculated the number of nucleotide differences (including deletions) between every pair of DNA sequences using the dist.dna program of the Ape package [38]. We then calculated the average proportion of nucleotide differences between species belonging to the same mammalian order and between species belonging to different orders. Second, we used the same approach to determine, for each mammalian order, how often two different species (or genera) have the exact same DNA sequence for the targeted region of the 16S rRNA gene.

### Targeted high-throughput sequencing of mammalian mt 16S rRNA genes and human mt genome hypervariable region I haplotypes

To interrogate the mammalian species composition of individual mosquito blood meals we amplified a 140 bp of the mammalian mt 16S rRNA gene using universal mammalian primers [36] modified to include a 5’-end tail complementary to the Illumina sequencing primers (S1 Table). We also attempted to amplify a subset of 192 mosquitoes with universal avian primers ([39] and S1 Fig) using a pooled approach but failed to detect any bird DNA in these samples. To identify individual differences among human blood meals, we designed PCR primers to amplify 300 bp of the human mt hypervariable region I. We first aligned 795 whole mt genomes of individuals from Oceania [40] using MAFFT version 7 [41] to evaluate the extent of sequence variation across the mt genome hypervariable region I and then designed primers positioned in conserved flanking sequences with Primer3 [42]. As described above, we added a 5’ tail to each primer for sample barcoding and high-throughput sequencing (S1 Table).

**Fig 1:**
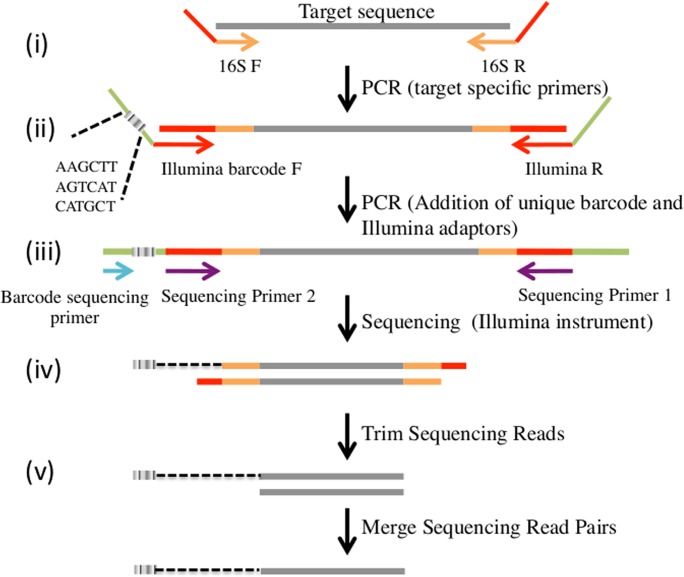
Overview of the sequencing assay used to characterize blood meal composition of individual mosquitoes. (i) A first PCR amplification is performed on DNA extracted from each mosquito targeting ~140 bp of the mammalian mt 16S rRNA (gray) using primers modified with a 5’-end tail complementary to the Illumina sequencing primers (red). (ii) A second PCR amplification incorporates the Illumina adaptors and a 6-nucleotide barcode unique to each mosquito at the ends of the individual blood meal PCR products. (iii) After pooling amplification products from 96 samples together, the PCR products are simultaneously sequenced on an Illumina MiSeq to (iv) generate paired-end reads (in grey) and barcode sequences (grey box). (v) Paired-end reads are then merged to provide error-corrected consensus sequence reads. The dotted black line indicates that the 6-nucleotide barcode corresponding to each read is known but is sequenced independently.

For each sample and amplicon, we performed two rounds of PCR amplification to prepare products for Illumina sequencing (Fig 1). First, we performed a locus-specific amplification (i.e., targeting either the mammalian mt 16S rRNA or the human mt hypervariable region) using the Promega GoTaq PCR kit protocol (50 μL reaction) with 1μL of genomic DNA, 0.2mM of each dNTP, 1.25 units of GoTaq DNA polymerase, 4mM of magnesium and 0.2 μM primers. PCR amplification was carried out under the following conditions: 3 minutes at 94°C followed by 30 cycles at 94°C for 45 seconds, 50°C for 45 seconds, 72°C for 30 seconds and a final elongation step at 72°C for 3 minutes. We then purified these PCR products using the QIAquick 96 PCR purification kit protocol (QIAGEN). Second, we incorporated Illumina adaptors, including unique 6-nucleotide sample identification barcode sequence through 10 additional PCR cycles, using barcoding primers complementary to the 5’-end tail incorporated during the first PCR amplification (Fig 1). For these reactions, we used the Promega GoTaq protocol as described above with 1uL of PCR product being added to each reaction. The same thermocycling conditions as described above were used but for an annealing temperature of 56°C. Predicted sizes for the mammalian mt 16S rRNA amplicons ranged from 265 to 343 bp; sizes for the human mt hypervariable region I amplicons ranged from 440 to 444 bp (amplicon sizes include Illumina sequencing primers, unique barcode sequence and Illumina adaptors, Fig 1). Finally, we pooled the barcoded amplification products from 96 individual mosquitoes and simultaneously sequenced them on an Illumina MiSeq instrument (Sequences deposited in NCBI SRA: SRP062959).

### Bioinformatic assessment of blood meal composition from individual mosquitoes

We discarded from further analyses any read that did not carry the exact barcode and primer sequences. After recording the read origin using the barcode sequence, we removed the primer and barcode sequences to only keep the amplified DNA sequences. We discarded any resulting read smaller than 50 bp as these likely represent primer dimers. Since each amplified molecule was sequenced in both directions using paired-end reads, we merged each pair of sequencing reads using PANDAseq [43] (Fig 1) keeping, at each position, the nucleotide with the highest sequencing quality. We then analyzed 16S RNA and human mtDNA sequences separately.

Using all 43,743,363 16S rRNA sequences generated from the 442 mosquitoes, we identified all unique DNA sequences using Mothur [44] and recorded the number of reads carrying each unique DNA sequence. We removed any DNA sequence that was observed less than 10 times across all samples, as these likely resulted from sequencing errors. We compared the remaining unique DNA sequences against all DNA sequences present in the NCBI nucleotide database using blastn. For each DNA sequence, we recorded the best match(es), only considering sequences with > 90% identity over the entire sequence length. We then retrieved the taxonomic information from each best-matched sequence using the ‘get_taxonomy’ function in PrimerTree. When an amplified sequence matched multiple species equally well, we recorded all species names associated with that sequence. We then summarized the blood meal of each mosquito by calculating the proportion of reads matching each species. As a small number of reads generated could reflect low level PCR contamination or an error in the sequence barcode identification, we only analyzed mosquito samples with at least 1,000 reads (S2 Fig). For the same reason, we considered a mosquito as having fed on a single mammalian host if >90% of the sequencing reads aligned to the 16S rRNA of that species. Alternatively, if >10% of the sequencing reads aligned to a second species, we considered the mosquito to have fed on multiple mammalian hosts.

For the human mt hypervariable region, we aligned the consensus reads to the human mitochondrial reference genome sequence (NC_012920.1) using bowtie2 [45] and calculated, for each sample, the number of reads supporting each haplotype. Only haplotypes supported by more than 500 reads were considered to avoid incorporating sequencing or PCR errors (i.e., rare haplotypes that differed from an abundant haplotype by one nucleotide difference) in the analyses. Finally, we reconstructed a phylogenetic tree with all identified human mt haplotypes using MEGA version 6 [46].

## Results

### *In silico* assessment of the amplification range and specificity of the universal mammalian 16S rRNA gene primer pairs

We first conducted extensive *in silico* analyses to confirm that the primer pair selected [36] could amplify DNA sequences from a wide range of mammalian orders including Primates, Rodentia (rodents), Artiodactyla (even-toed ungulates), Carnivora (carnivorans), Chiroptera (bats), Cetacea (cetaceans), Insectivora (insectivores) and Marsupials (Table 1). Overall, *in silico* analysis predicted that these primers should amplify 1,752 of the 1,779 mammalian species (98.5%) sequenced at this locus and present in the NCBI nucleotide database (Table 1). Besides mammals, these primers were predicted to also amplify Actinopteri (bony-fishes) and Amphibia (amphibians) (S3 Fig).

**Table 1:** Summary of the amplification range and discriminatory power predicted for the mammalian 16S rRNA primers. The table indicates, for each mammalian order, the number of species deposited in NCBI for the 16S rRNA genes, the number of species predicted to be amplified by the universal primers as well as the percentage of genera and species that would carry a unique sequence for this locus (enabling their rigorous identification).

	Orders*	# of species	# species amplified	Genus	Species
Placental					
	Artiodactyla (even-toed ungulates)	209	206	93.6	76.1
	Carnivora (carnivores)	141	138	97	86.1
	Cetacea (whales)	73	73	58.1	44.2
	Chiroptera (bats)	423	421	97.7	89.8
	Insectivora (insectivores)	174	169	100	84.8
	Lagomorpha (rabbits and hares)	24	21	100	68.4
	Macroscelidea (elephant shrews)	12	12	100	100
	Perissodactyla (odd-toed ungulates)	22	21	100	68.4
	Primates (primates)	195	192	100	94.6
	Rodentia (rodents)	404	400	100	91.2
	Scandentia (tree shrews)	19	17	100	88.2
Marsupial					
	Dasyuromorphia (quolls, dunnarts, and numbats)	66	67	100	90.9
	Didelphimorphia (opposums)	19	19	89.5	89.5
	Diprotodontia (possums, kangaroos, and wallabies)	63	62	100	100
	Peramelemorphia (bandicoots and bilbies)	14	14	100	85.7

*This table does not include orders for which less than 10 sequences were available in NCBI for this locus.

In addition to amplifying a wide range of targets, our approach requires primers to amplify DNA sequences containing enough information to identify each species specifically. We tested this parameter by comparing the DNA sequences predicted to be amplified by this primer pair (see Methods for details). Despite the short amplified DNA sequence (~140 bp), these primers enabled rigorous differentiation of most mammalian species as illustrated by the average proportion of nucleotide differences (including deletions) between sequences of species belonging to the same or different order (S2 Table). For example, 27% of the nucleotide positions at this locus differ, on average, between one Carnivora and one Primate species and 17% of the nucleotides differ between the sequences of two Carnivora species. This high discriminating ability is also shown by the long branch lengths displayed by the phylogenetic tree reconstructed using these sequences (S1C Fig). In fact, we found that one DNA sequence amplified by these primers typically matches a single genus and, in 86% of the cases, a single species (Table 1).

### Application to field-caught female *Anopheles* mosquito blood meals

We analyzed mosquitoes collected in five villages in the Madang Province in PNG: Dimer (n = 45), Wasab (n = 81), Kokofine (n = 83), Mirap (n = 171) and Matukar (n = 62). These mosquitoes included several species of the *Anopheles punctulatus* group: *An*. *punctulatus s*.*s*., *An*. *koliensis*, *An*. *farauti* 4 and *An*. *farauti s*.*s*. We characterized the blood meal composition of a total of 442 female *Anopheles* by amplifying the mammalian mt 16S rRNA genes from DNA extracted from these mosquitoes, pooling the PCR products of 96 samples after individual barcoding, and simultaneously sequencing the samples on an Illumina MiSeq instrument (Fig 1). We generated a total of 43,743,363 paired-end reads of 150 bp (includes added primers). For 42,198,573 DNA sequences (96.5%), we were able to collapse the overlapping paired-ends (Fig 1) and thus correct many sequencing errors. After combining the reads generated from all samples together, we identified 2,436,277 unique DNA sequences. We discarded from further analyses 2,404,684 unique DNA sequences that were carried by less than 10 reads across all samples as these likely represent DNA sequences with rare sequencing errors (accounting for a total of 4,432,784 reads or 10.1% of the total number of reads generated). We then compared the remaining 31,593 unique DNA sequences, accounting for 39,310,579 reads (89.9%), to all DNA sequences deposited in the NCBI database. 28,999 of these DNA sequences (representing 38,375,616 reads) had > 90% nucleotide identity to at least one mammalian DNA sequence present in NCBI: 18,814 unique DNA sequences best matched a single mammalian species sequence while 10,185 unique DNA sequences matched equally well to multiple mammalian species sequences (S3 Table).

Overall, we generated an average of 82,528 reads per mosquito. The number of reads generated from each mosquito varied considerably (S2 Fig) as it depends on several factors including: the amount of starting template (i.e., quantity of mammalian DNA present in the mosquito), the amplification efficiency and uneven pooling or variations in sequencing output between MiSeq runs. For further analyses, we only considered mosquito samples with more than 1,000 reads. None of the 30 extraction controls (i.e., water samples that have been processed in parallel through DNA extraction, PCR and sequencing) reached this cutoff illustrating the low level of cross-contamination or read mis-assignment due to errors in the barcode sequence (if any). Overall, we analyzed mammalian DNA from 314 blood fed mosquitoes, including 258 out of the 337 mosquitoes characterized as fully-fed (76.6%) and 56 out of the 86 mosquitoes visually-classified as partially-fed (65.1%). Only 5 out of the 19 mosquitoes visually classified as non-fed yielded mammalian 16S rRNA sequences: four yielded exclusively human 16S rRNA sequences, the last one a mix of human and pig sequences. These DNA sequences could indicate possible contamination either during field collection or in the laboratory, or detection of DNA from a previous, partially digested, blood meal. There was no statistical difference between fully-fed and partially-fed mosquitoes, however the number of sequencing reads generated for mosquitoes visually classified as fully-fed or partially-fed were significantly different from those classified as non-fed (p<0.05, Wilcoxon Rank-Sum test, S4 Fig). In total we successfully amplified and sequenced mammalian DNA from 319 *Anopheles* mosquitoes.

We identified 201 *Anopheles* mosquitoes that carried human DNA, 111 carried pig DNA, 60 carried dog DNA and 5 carried mouse DNA (Table 2; further details in S3 and S4 Tables). In addition to these expected hosts, we identified one mosquito that carried DNA from two different bat species: 7.2% of the reads matched perfectly *Dobsonia moluccenis*, a fruit bat commonly found in PNG, while 5.1% of the reads were most similar (94.4% identity) to another megabat species, *Dobsonia praedatrix*, also endemic to PNG (Table 2). These bat DNA sequences were clearly distinct (8 nucleotide differences between them) and unlikely to have been derived from sequencing errors, indicating that the mosquito fed on two different bats (S5 Fig). Additionally, in another mosquito 13% of the total reads (7,599 reads) were most similar to the common spotted cuscus (*Spilocuscus maculatus*, 98% similarity), a marsupial found in the forests of PNG (S6 Fig). Note that, consistent with our *in silico* analysis, we were not always able to identify the exact species that was fed upon. For example, we could not differentiate *Canis lupus* from *Canis aureus* (S3 Table). Overall, these finding illustrate the unbiased nature of this sequencing approach to identify host species regardless of expectations for mosquito blood meal feeding (as long as a closely related species has been sequenced).

**Table 2:** Summary of the hosts identified in the mosquito blood meals. For each host, the number of *Anopheles* mosquitoes carrying a corresponding DNA sequencing is indicated as well as the highest percent identity between the read generated and the host DNA sequence in NCBI and the average number of reads per sample carrying each DNA sequence.

Name	# samples detected	Percent Identity	Average # of reads
Human	201	100	71,971
Pig	111	100	75,273
Dog	60	100	83,412
Mouse	5	100	3,218
*Dobsonia moluccensis*	1	100	2,664
*Dobsonia praedatrix*	1	94.4	1,916
*Spilocuscus maculatus*	1	98	7,599

**Fig 2:**
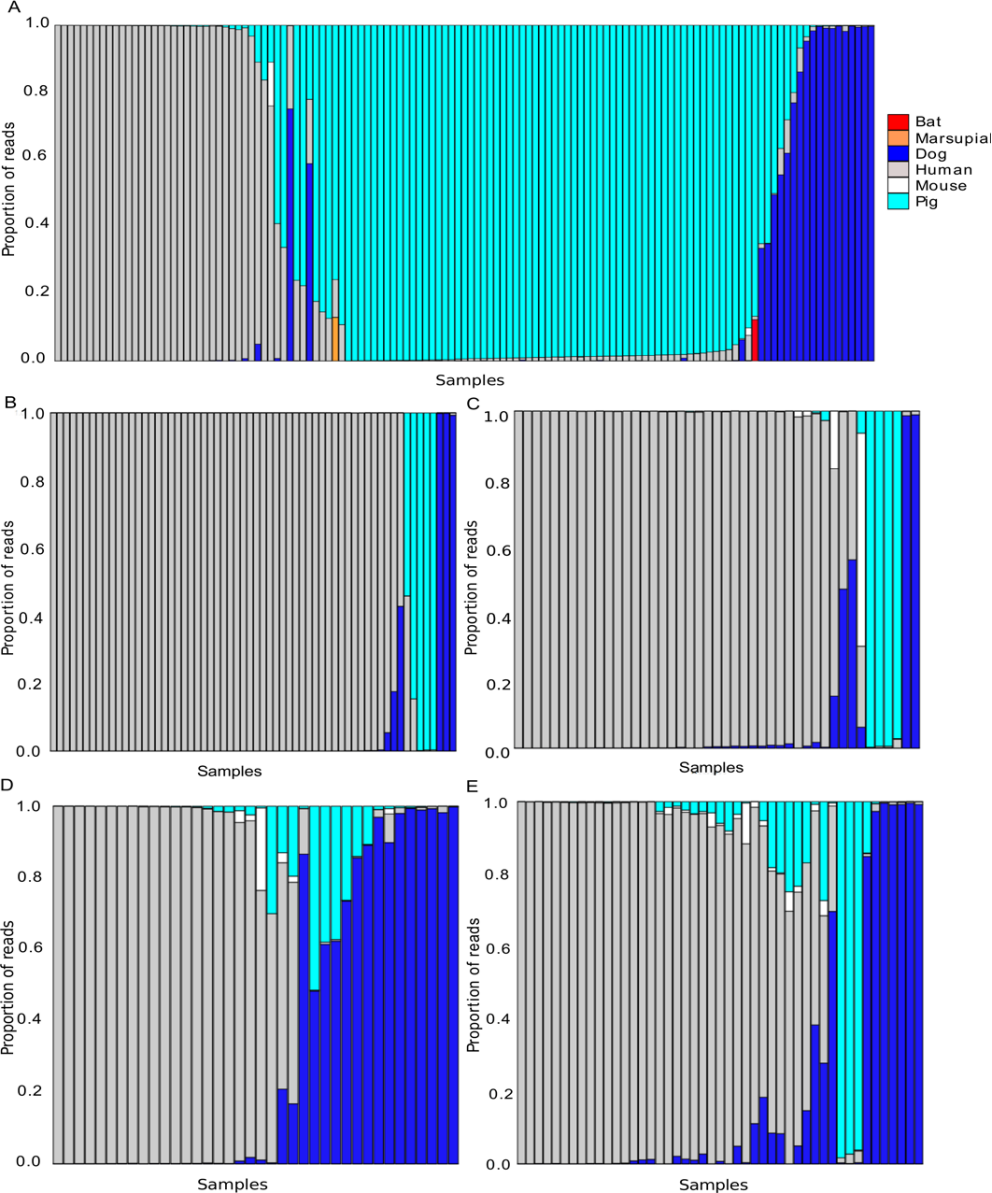
**Composition of the blood meals for mosquitoes collected in Mirap (A), Kokofine (B), Wasab (C), Dimer (D) and Matukar (E).** Each vertical bar shows the composition of the blood meal for one mosquito: each color represents a different host species and the height of each stacked bar corresponds to the proportion of reads matching this host DNA sequence. Gray corresponds to human DNA, turquoise to pig, blue to dog, white to mouse, red to bat and orange to cuscus.

Out of 319 mosquitoes analyzed, 52 (16.3%) showed clear evidence of having fed on more than one host species (with >10% of the reads supporting the minor host): 44 mosquitoes carried DNA from two species and eight carried DNA from three species (Fig 2).

Within each village, we identified three major mammalian hosts—humans, dogs and pigs—accounting for 37 to 100% of each mosquito blood meal. However, the proportion of mosquitoes that fed on each host varied within and between villages (Fig 2). For example, in Mirap, only 31 of the 127 *Anopheles* mosquitoes (24%) fed on humans while 62 (49%) fed on pigs, 11 fed on dogs (9%) and 23 fed on two or three species (18%) including one mosquito that fed on two bat species and one mosquito that fed on a common spotted cuscus (Fig 2A). By contrast, in Kokofine, 52 out of the 62 mosquitoes fed on humans (84%) while the remaining 10 mosquitoes fed on dogs (n = 3), pigs (n = 3) or on multiple species (n = 4) (Fig 2B). The data for the three other villages are presented in Fig 2C–2E. Note that as host density information is not available for these villages, we were unable to test whether the observed differences in blood meal composition were caused by differences in mosquito feeding behavior among locations or species.

### Evidence of mosquito blood meals containing multiple human hosts

**Fig 3:**
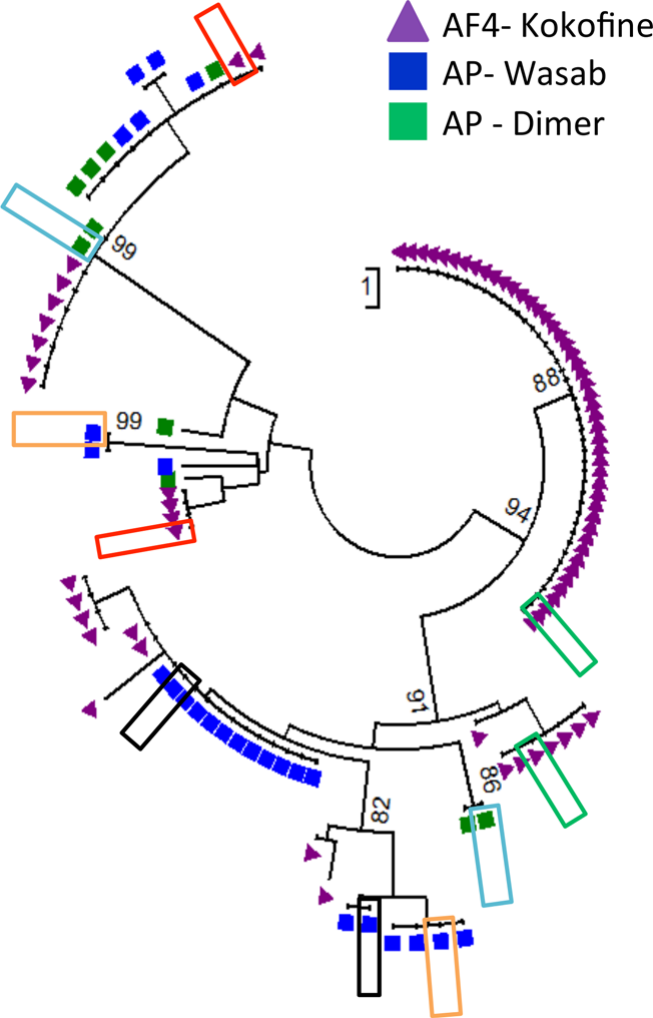
Neighbor-joining tree showing the relationships among the human mtDNA haplotypes amplified from mosquitoes. Each symbol represents one DNA sequence amplified from one mosquito. Different shapes represent different *Anopheles* species (squares-*An*. *punctulatus s*.*s*., triangles-*An*. *farauti* 4) and is colored according to the collection site (green-Dimer, blue-Wasab, purple-Kokofine). Mixed blood meals are highlighted by boxes of the same color: for example, the two red boxes show two human mtDNA haplotypes amplified from a single *An*. *farauti* 4 mosquito collected in Kokofine.

Since we observed that 16.3% of the mosquitoes analyzed had fed on multiple mammalian hosts, we hypothesized that mosquitoes could also be feeding on multiple human individuals. We therefore investigated the number of different human DNA sequences present in 157 human-fed mosquitoes, using the same approach to sequence ~300 bp of the human mt hypervariable region. We generated an average of 26,721 sequencing reads of 250 bp for each sample and successfully amplified 102 of the 157 mosquitoes for the human mt hypervariable regions yielding a total of 20 different human mtDNA sequences (S7 Fig). While a single DNA sequence was amplified from 78.5% (n = 80) of the human-fed mosquitoes analyzed, 21.5% (n = 22) mosquitoes carried two distinct DNA sequences (S7 Fig). One sequence, identified in 14 of these potential mixed human blood meal, was always present at low frequency (<8% of the reads) and was actually more similar to a region of human chromosome 11 (98% similarity) than to the mitochondrial genome sequence (91%). This DNA sequence likely resulted from the amplification of the nuclear insertion of the mitochondrial sequence (numt, [47]) and was excluded from further analyses. Nine mosquitoes, belonging to two species and collected in three locations, showed presence of two human mtDNA sequences (S5 Table). For four of these mosquitoes, only one substitution (out of the 300 bp amplified) differentiated the two sequences and these could possibly be caused by a PCR error occurring at an early cycle. However, for the remaining five mosquitoes, 5–14 nucleotide substitutions differentiated the two sequences amplified and indicated that the mosquito successively fed on multiple individuals (Fig 3 and S5 Table).

## Discussion

Vector-borne diseases such as dengue, malaria, Chagas disease or leishmaniasis, account for more than 17% of all human infectious diseases and cause more than one million deaths annually [48]. To control and eliminate these diseases, it is essential that we fully appreciate the diversity and relative importance of the disease hosts and vectors. For example, while birds are well known to be the primary reservoir host of Eastern Equine Encephalitis virus (EEEV), a virus transmitted by mosquitoes that can cause zoonotic infections, recent studies have shown that snakes constitute another, previously unsuspected, reservoir of EEEV [49].

Most molecular techniques used to investigate insects’ blood meal composition are specifically designed to identify one or a few specific host(s) and cannot characterize blood meal composition in an agnostic manner. Universal primer pairs have been used to circumvent this limitation and amplify any mammalian [10, 50], or vertebrate DNA [51, 52]. However, these former studies have relied on cloning the amplified products and sequencing a few clones from each insect and are consequently very expensive and labor intensive. In addition, the presence of multiple host species in a blood meal complicates the sequence analysis when the amplification product is sequenced directly (resulting in high background noise) or further increases the cost of the experiment if the PCR products are cloned and several clones sequenced per mosquito. These challenges have limited the number of studies that rigorously examined mixed blood meals from disease vectors and provided a potentially incomplete perspective on these vectors’ feeding patterns. Rigorous identification of mixed blood meals is however critical to understand disease transmission as it might reveal higher transmission rates, if a blood meal typically consist of the blood from multiple individuals, or, lower, if the insect often feeds on species not susceptible to infection.

By contrast, a unique strength of the assay described here is its ability to rigorously detect and quantify mixed blood meals by identifying, in a single mosquito, the presence of multiple species’ DNA even if they only contribute to a small fraction of the entire blood meal (down to 10% in the current study). We were able to accurately detect and quantify mixed blood meals due to the high sequencing coverage achieved by high-throughput sequencing: on average, mammalian mt 16S rRNA genes amplified from each mosquito was sequenced by 82,528 reads and, therefore, even minor host DNA present in 10% of the total mammalian DNA was represented by several thousand reads. Note that the DNA amplification might have different efficiency for different DNA sequences (e.g., amplify better pig than dog and human DNA). Consequently, the proportion of reads obtained from each species might not reflect the true proportions of these species in the blood meal (especially since the mtDNA content in blood might also vary among species). However, this possible bias will affect all samples similarly and will not interfere with comparisons of the blood meal composition across samples. In addition the host DNA is degraded after the blood meal and the time between the mosquito’s meal and sample collection could therefore influence the interpretation of the results. Note that in mosquitoes, host blood meals can typically be detected up to 24–30 hours post-feeding, but have been detected up to 48 hours post-feeding [8].

The second key feature of our approach is its ability to detect novel blood hosts that would not have been detected using traditional techniques. For example, here we report the first observation that *Anopheles* mosquitoes can feed on bats and marsupials. Importantly, all the hosts identified in our study are endemic to New Guinea where our samples were collected. For one of the bat sequences, we were not able to identify the exact species (as the most similar sequence in NCBI only had 94.4% identity) but our analyses revealed that it is likely closely related to the megabat *Dobsonia praedatrix* (1,916 sequence reads). This result also illustrates that, even if the actual host has not been sequenced for the locus of interest, our approach can still reveal its presence (and guide future studies to obtain more precise taxonomic information).

There are however some limitations to this approach. First, the primers may not allow the exact species to be identified: we estimated that 14% of the mammalian species do not have a unique DNA sequence at the locus amplified and the sequencing may therefore not enable differentiation among several closely related species. However, this limitation could easily be overcome by designing species-specific primers for a more variable region (e.g., the mt hypervariable region). Second, since we are comparing DNA sequences to the NCBI nt database there is the possibility of identifying incorrectly annotated sequences or pseudogenes, which could introduce spurious results. For example, one of the DNA sequences amplified that matched perfectly many pig DNA sequences (*Sus scrofa*, *Sus barbatus*, *Sus philippensis*, *Sus celebensis and Sus verrucosus*) was also identical to a thrip DNA sequence (*Scolothrips takahashii)*. This instance likely represents a misannotation in NCBI but could be problematic without stringent quality controls. Similarly, several DNA sequences matched equally well human and gorilla, chimpanzee or orangutan DNA sequences and likely represent amplification of nuclear pseudogenes (numt) common in apes. Typically, these sequences were supported by a much lower number of reads (on average, 411) than DNA sequences that perfectly matched *Homo sapiens* mtDNA (on average represented by 70,405 reads) (S3 Table). Lastly, given the sensitivity of PCR and of the sequencing detection method, it is important that stringent controls are used to rule out human contamination. Here, we included 30 extraction (water) controls that were all negative suggesting very low levels of laboratory contamination (if any). An interesting complementary control, which would also control for field contamination, would be to analyze male mosquitoes collected at the same time.

Finally, our approach enables simultaneous processing of batches of 96 samples with minimum hands-on time (7–9 hours of laboratory work). This provides a unique throughput that is essential to analyze several hundred mosquitoes for well-powered comparisons. In addition, the high multiplexing of our approach dramatically reduces the cost of next-generation sequencing (to less than US$10 per sample), especially when combining the characterization of the blood meal composition with other data such as intra-species host characterization (see below), molecular species determination or genotyping.

### DNA profiling of human maternal lineages from field collected mosquitoes

Previous studies have used microsatellites to compare the attractiveness of different individuals or group of individuals [22, 24, 25], examine the blood feeding patterns of mosquitoes [20, 53, 54] or determine the effectiveness of insecticide treated bed nets [55–58]. DNA profiling with microsatellites allows for the identification of unique genetic profiles from human individuals fed on and can be a very powerful method to differentiate DNA from unrelated individuals. However, microsatellites can only detect the simultaneous presence of multiple individual DNAs (typically two) if their proportion in one sample is relatively similar. Otherwise, the signal from the less abundant DNA is typically obscured and not distinguishable from background noise. Rigorously identifying whether a disease vector feeds on a single or multiple individuals is however essential for disease control as vectors that feed on multiple individuals are more likely to rapidly spread the disease than those that only feed on a single individual.

As an alternative to microsatellites, our approach relies on identifying unique human mitochondrial haplotypes carried by a mosquito by analyzing 300 bp of the mt hypervariable region I. We showed that at least five (out of 102 mosquitoes analyzed) carried human mitochondrial DNA sequences from more than one person. It is important to emphasize here that the number of mixed human blood meals is clearly underestimated as only maternal lineages can be detected by this approach: all offspring will carry the same DNA sequence as their mother and therefore it would not be possible to distinguish between siblings (or cousins from mothers who are sisters). However, one could, at least partially, circumvent this limitation by including additional polymorphic nuclear loci in the assay and sequence them together with the mt hypervariable region locus (and the 16S rRNA). Overall, our approach allows for a rapid evaluation of the number of maternal lineages a mosquito has fed on that can be added to the characterization of the blood meal at no additional costs, and could be used to determine if mosquitoes preferentially feed on some individuals and avoid other individuals.

## Supporting Information

S1 Table Primers used in this study to amplify mammalian host blood meals and the human mitochondrial hypervariable region I. (XLSX)

S2 Table Proportion of nucleotide differences, including deletions, between sequences of species in the same or different mammalian order. (XLSX)

S3 Table Summary of blast results showing, for each species identified, the average number of corresponding reads per sample, the number of mosquito samples that carried a DNA sequence matching this species and the maximum percent identity between the reads and NCBI sequence. Note that when the sequences generated matched several species equally well, these are all indicated.
(XLSX)

S4 Table Summary of mammalian blood hosts fed on showing, for each sample, the collection site, mosquito species, number of sequencing reads matching each mammalian blood host and the total number of sequencing reads generated. (XLSX)

S5 Table Mixed human blood meals. The table shows, for each mosquito with multiple human mtDNA sequences, the collection site, the mosquito species, the number of nucleotide differences between the two mtDNA sequence and the proportion of the minor sequence.
(XLSX)

S1 Fig Neighbor-joining tree reconstructed using the DNA sequences predicted to be amplified by primers targeting the mammalian mitochondrial (A) COI, (B) Cytb [37] and (C) 16S rRNA [36] as well as by primers targeting the (D) avian 12S ribosomal RNA [39]. Each colored dot represents a different DNA sequence that is colored according to its taxonomy. Despite being much smaller (140 bp on average vs 704 bp for COI and 819 bp for Cyt B), the 16S rRNA sequences provides similar information content as the other mitochondrial genes. (Note that the short length of the 16S rRNA amplicon greatly facilitates next-generation sequencing). In addition, the number of mammalian species that have been sequenced for 16S rRNA (N = 1,752) is much greater than for the other loci (respectively, 244 and 225 for COI and CytB) enabling more robust species identification.
(PDF)

S2 Fig Summary of the sequencing depth for each mosquito sample. Each vertical bar represents a single mosquito ranked along the x-axis according to the number of reads obtained to characterize its blood meal (y-axis, log scaled). The panel underneath the plot indicates whether the mosquitoes were visually classified as fed (fully-fed and partially-fed, green horizontal bar) or non-fed (blue bar). Extraction controls (water) are represented by the black horizontal bar. The horizontal red bar at 1,000 indicates the cut-off used for analysis inclusion.
(PDF)

S3 Fig Neighbor-joining tree reconstructed using the DNA sequences predicted to be amplified by the mammalian mt 16S rRNA primers. Each colored dot represents a different DNA sequence. The tree shows the entire range of species amplified and colored by classes (Blue, mammals; Red, bony-fish; Green, amphibians).
(PDF)

S4 Fig Box plot showing the number of sequencing reads generated per mosquito according to the blood meal status of mosquitoes determined visually (only samples from one sequencing run are displayed). (PDF)

S5 Fig Neighbor-joining phylogenetic tree showing the species relationships among bat species based on the DNA sequence targeted with the 16S mt rRNA primers. The two bat DNA sequences amplified from one mosquito’s blood meal are shown by the red boxes.
(PDF)

S6 Fig Neighbor-joining phylogenetic tree showing the relationships among marsupial genera based on the DNA sequence targeted with the 16S rRNA mt primers. The marsupial DNA sequence amplified from one mosquito’s blood meal is shown in the red box.
(PDF)

S7 Fig Neighbor-joining tree showing the relationships among human DNA sequences amplified using the mt hypervariable primers. The shapes indicate the species of each mosquito carrying a specific human DNA sequence (squares represent *An*. *punctulatus s*.*s*., triangles *An*. *farauti* 4). The color of each shape indicates the village where the mosquito was collected (green from Dimer, blue from Wasab, and purple from Kokofine). Note the long-branch separating the mitochondrial DNA sequences from the nuclear insertion (numt) sequence.
(PDF)

## References

[1] Lounibos LP. Invasions by insect vectors of human disease. Annual review of entomology. 2002;47:233–66. 10.1146/annurev.ento.47.091201.145206 . 11729075

[2] Gratz NG. Emerging and resurging vector-borne diseases. Annual review of entomology. 1999;44:51–75. 10.1146/annurev.ento.44.1.51 . 9990716

[3] Tempelis CH. Host-feeding patterns of mosquitoes, with a review of advances in analysis of blood meals by serology. Journal of medical entomology. 1975;11(6):635–53. . 235647 10.1093/jmedent/11.6.635

[4] Washino RK, Tempelis CH. Mosquito host bloodmeal identification: methodology and data analysis. Annual review of entomology. 1983;28:179–201. 10.1146/annurev.en.28.010183.001143 . 6131641

[5] Beier JC, Perkins PV, Wirtz RA, Koros J, Diggs D, Gargan TP 2nd, et al Bloodmeal identification by direct enzyme-linked immunosorbent assay (ELISA), tested on Anopheles (Diptera: Culicidae) in Kenya. Journal of medical entomology. 1988;25(1):9–16. . 3357176 10.1093/jmedent/25.1.9

[6] Lardeux F, Loayza P, Bouchite B, Chavez T. Host choice and human blood index of Anopheles pseudopunctipennis in a village of the Andean valleys of Bolivia. Malaria journal. 2007;6:8 10.1186/1475-2875-6-8 17241459 PMC1783659

[7] Kent RJ. Molecular methods for arthropod bloodmeal identification and applications to ecological and vector-borne disease studies. Molecular ecology resources. 2009;9(1):4–18. 10.1111/j.1755-0998.2008.02469.x . 21564560

[8] Kent RJ, Norris DE. Identification of mammalian blood meals in mosquitoes by a multiplexed polymerase chain reaction targeting cytochrome B. The American journal of tropical medicine and hygiene. 2005;73(2):336–42. 16103600 PMC4147110

[9] Ngo KA, Kramer LD. Identification of mosquito bloodmeals using polymerase chain reaction (PCR) with order-specific primers. Journal of medical entomology. 2003;40(2):215–22. . 12693851 10.1603/0022-2585-40.2.215

[10] Hamer GL, Kitron UD, Brawn JD, Loss SR, Ruiz MO, Goldberg TL, et al Culex pipiens (Diptera: Culicidae): a bridge vector of West Nile virus to humans. Journal of medical entomology. 2008;45(1):125–8. . 18283952 10.1603/0022-2585(2008)45[125:cpdcab]2.0.co;2

[11] Crabtree MB, Kading RC, Mutebi JP, Lutwama JJ, Miller BR. Identification of host blood from engorged mosquitoes collected in western Uganda using cytochrome oxidase I gene sequences. Journal of wildlife diseases. 2013;49(3):611–26. 10.7589/2012-08-213 . 23778610

[12] Allan BF, Goessling LS, Storch GA, Thach RE. Blood meal analysis to identify reservoir hosts for Amblyomma americanum ticks. Emerging infectious diseases. 2010;16(3):433–40. 10.3201/eid1603.090911 20202418 PMC3322017

[13] Che Lah EF, Yaakop S, Ahamad M, Md Nor S. Molecular identification of blood meal sources of ticks (Acari, Ixodidae) using cytochrome b gene as a genetic marker. ZooKeys. 2015;(478):27–43. 10.3897/zookeys.478.8037 25685009 PMC4319051

[14] Pichon B, Egan D, Rogers M, Gray J. Detection and identification of pathogens and host DNA in unfed host-seeking Ixodes ricinus L. (Acari: Ixodidae). Journal of medical entomology. 2003;40(5):723–31. 14596289 10.1603/0022-2585-40.5.723

[15] Valinsky L, Ettinger G, Bar-Gal GK, Orshan L. Molecular identification of bloodmeals from sand flies and mosquitoes collected in Israel. Journal of medical entomology. 2014;51(3):678–85. . 24897862 10.1603/me13125

[16] Haouas N, Pesson B, Boudabous R, Dedet JP, Babba H, Ravel C. Development of a molecular tool for the identification of Leishmania reservoir hosts by blood meal analysis in the insect vectors. The American journal of tropical medicine and hygiene. 2007;77(6):1054–9. . 18165521

[17] Soares VY, Silva JC, Silva KR, Pires e Cruz Mdo S, Santos MP, Ribolla PE, et al Identification of blood meal sources of Lutzomyia longipalpis using polymerase chain reaction-restriction fragment length polymorphism analysis of the cytochrome B gene. Memorias do Instituto Oswaldo Cruz. 2014;109(3):379–83. 24821056 10.1590/0074-0276130405 PMC4131795

[18] Steuber S, Abdel-Rady A, Clausen PH. PCR-RFLP analysis: a promising technique for host species identification of blood meals from tsetse flies (Diptera: Glossinidae). Parasitology research. 2005;97(3):247–54. 10.1007/s00436-005-1410-y . 15999278

[19] Muturi CN, Ouma JO, Malele II, Ngure RM, Rutto JJ, Mithofer KM, et al Tracking the feeding patterns of tsetse flies (Glossina genus) by analysis of bloodmeals using mitochondrial cytochromes genes. PloS one. 2011;6(2):e17284 10.1371/journal.pone.0017284 21386971 PMC3046180

[20] Norris LC, Fornadel CM, Hung WC, Pineda FJ, Norris DE. Frequency of multiple blood meals taken in a single gonotrophic cycle by Anopheles arabiensis mosquitoes in Macha, Zambia. The American journal of tropical medicine and hygiene. 2010;83(1):33–7. 10.4269/ajtmh.2010.09–0296 20595474 PMC2912572

[21] Chow-Shaffer E, Sina B, Hawley WA, De Benedictis J, Scott TW. Laboratory and field evaluation of polymerase chain reaction-based forensic DNA profiling for use in identification of human blood meal sources of Aedes aegypti (Diptera: Culicidae). Journal of medical entomology. 2000;37(4):492–502. . 10916289 10.1603/0022-2585-37.4.492

[22] Ansell J, Hamilton KA, Pinder M, Walraven GE, Lindsay SW. Short-range attractiveness of pregnant women to Anopheles gambiae mosquitoes. Transactions of the Royal Society of Tropical Medicine and Hygiene. 2002;96(2):113–6. . 12055794 10.1016/s0035-9203(02)90271-3

[23] Michael E, Ramaiah KD, Hoti SL, Barker G, Paul MR, Yuvaraj J, et al Quantifying mosquito biting patterns on humans by DNA fingerprinting of bloodmeals. The American journal of tropical medicine and hygiene. 2001;65(6):722–8. . 11791964 10.4269/ajtmh.2001.65.722

[24] Scott TW, Githeko AK, Fleisher A, Harrington LC, Yan G. DNA profiling of human blood in anophelines from lowland and highland sites in western Kenya. The American journal of tropical medicine and hygiene. 2006;75(2):231–7. . 16896124

[25] De Benedictis J, Chow-Shaffer E, Costero A, Clark GG, Edman JD, Scott TW. Identification of the people from whom engorged Aedes aegypti took blood meals in Florida, Puerto Rico, using polymerase chain reaction-based DNA profiling. The American journal of tropical medicine and hygiene. 2003;68(4):437–46. . 12875293

[26] Replogle J, Lord WD, Budowle B, Meinking TL, Taplin D. Identification of host DNA by amplified fragment length polymorphism analysis: preliminary analysis of human crab louse (Anoplura: Pediculidae) excreta. Journal of medical entomology. 1994;31(5):686–90. . 7966171 10.1093/jmedent/31.5.686

[27] Mumcuoglu KY, Gallili N, Reshef A, Brauner P, Grant H. Use of human lice in forensic entomology. Journal of medical entomology. 2004;41(4):803–6. . 15311479 10.1603/0022-2585-41.4.803

[28] Beebe NW, Russell T, Burkot TR, Cooper RD. Anopheles punctulatus group: evolution, distribution, and control. Annual review of entomology. 2015;60:335–50. 10.1146/annurev-ento-010814-021206 . 25341094

[29] Beebe NW, Cooper RD. Distribution and evolution of the Anopheles punctulatus group (Diptera: Culicidae) in Australia and Papua New Guinea. International journal for parasitology. 2002;32(5):563–74. . 11943229 10.1016/s0020-7519(01)00359-9

[30] Charlwood JD, Dagoro H, Paru R. Blood-feeding and resting behaviour in the Anopheles punctulatus Donitz complex (Diptera: Culicidae) from coastal Papua New Guinea. Bulletin of Entomological Research. 1985;75(3):463–76. 10.1017/S0007485300014577.

[31] Burkot TR, Graves PM, Paru R, Lagog M. Mixed blood feeding by the malaria vectors in the Anopheles punctulatus complex (Diptera: Culicidae). Journal of medical entomology. 1988;25(4):205–13. . 3404539 10.1093/jmedent/25.4.205

[32] Burkot TR, Russell TL, Reimer LJ, Bugoro H, Beebe NW, Cooper RD, et al Barrier screens: a method to sample blood-fed and host-seeking exophilic mosquitoes. Malaria journal. 2013;12:49 10.1186/1475-2875-12-49 23379959 PMC3574015

[33] Belkin J. The Mosquitoes of the South Pacfic (Diptera: Culicidae). Berkeley, CA: University of California Press; 1962.

[34] Logue K, Small ST, Chan ER, Reimer L, Siba PM, Zimmerman PA, et al Whole-genome sequencing reveals absence of recent gene flow and separate demographic histories for Anopheles punctulatus mosquitoes in Papua New Guinea. Molecular ecology. 2015;24(6):1263–74. 10.1111/mec.13107 . 25677924 PMC4504211

[35] Henry-Halldin CN, Reimer L, Thomsen E, Koimbu G, Zimmerman A, Keven JB, et al High throughput multiplex assay for species identification of Papua New Guinea malaria vectors: members of the Anopheles punctulatus (Diptera: Culicidae) species group. The American journal of tropical medicine and hygiene. 2011;84(1):166–73. 10.4269/ajtmh.2011.10–0438 21212222 PMC3005508

[36] Taylor PG. Reproducibility of ancient DNA sequences from extinct Pleistocene fauna. Molecular biology and evolution. 1996;13(1):283–5. . 8583902 10.1093/oxfordjournals.molbev.a025566

[37] Navia-Gine WG, Loaiza JR, Miller MJ. Mosquito-host interactions during and after an outbreak of equine viral encephalitis in Eastern Panama. PloS one. 2013;8(12):e81788 10.1371/journal.pone.0081788 24339965 PMC3858258

[38] Paradis E, Claude J, Strimmer K. APE: Analyses of Phylogenetics and Evolution in R language. Bioinformatics. 2004;20(2):289–90. . 14734327 10.1093/bioinformatics/btg412

[39] Epp LS, Boessenkool S, Bellemain EP, Haile J, Esposito A, Riaz T, et al New environmental metabarcodes for analysing soil DNA: potential for studying past and present ecosystems. Molecular ecology. 2012;21(8):1821–33. 10.1111/j.1365-294X.2012.05537.x . 22486821

[40] Duggan AT, Evans B, Friedlaender FR, Friedlaender JS, Koki G, Merriwether DA, et al Maternal history of Oceania from complete mtDNA genomes: contrasting ancient diversity with recent homogenization due to the Austronesian expansion. American journal of human genetics. 2014;94(5):721–33. 10.1016/j.ajhg.2014.03.014 24726474 PMC4067553

[41] Katoh K, Standley DM. MAFFT multiple sequence alignment software version 7: improvements in performance and usability. Molecular biology and evolution. 2013;30(4):772–80. 10.1093/molbev/mst010 23329690 PMC3603318

[42] Untergasser A, Cutcutache I, Koressaar T, Ye J, Faircloth BC, Remm M, et al Primer3—new capabilities and interfaces. Nucleic acids research. 2012;40(15):e115 10.1093/nar/gks596 22730293 PMC3424584

[43] Masella AP, Bartram AK, Truszkowski JM, Brown DG, Neufeld JD. PANDAseq: paired-end assembler for illumina sequences. BMC bioinformatics. 2012;13:31 10.1186/1471-2105-13-31 22333067 PMC3471323

[44] Schloss PD, Westcott SL, Ryabin T, Hall JR, Hartmann M, Hollister EB, et al Introducing mothur: open-source, platform-independent, community-supported software for describing and comparing microbial communities. Applied and environmental microbiology. 2009;75(23):7537–41. 10.1128/AEM.01541-09 19801464 PMC2786419

[45] Langmead B, Salzberg SL. Fast gapped-read alignment with Bowtie 2. Nature methods. 2012;9(4):357–9. Epub 2012/03/06. 10.1038/nmeth.1923 22388286 PMC3322381

[46] Tamura K, Stecher G, Peterson D, Filipski A, Kumar S. MEGA6: Molecular Evolutionary Genetics Analysis version 6.0. Molecular biology and evolution. 2013;30(12):2725–9. 10.1093/molbev/mst197 24132122 PMC3840312

[47] Leister D. Origin, evolution and genetic effects of nuclear insertions of organelle DNA. Trends in genetics: TIG. 2005;21(12):655–63. 10.1016/j.tig.2005.09.004 . 16216380

[48] WHO. Vector-borne diseases Fact sheet March 2014. Available from: http://www.who.int/mediacentre/factsheets/fs387/en/.

[49] Graham SP, Hassan HK, Chapman T, White G, Guyer C, Unnasch TR. Serosurveillance of eastern equine encephalitis virus in amphibians and reptiles from Alabama, USA. The American journal of tropical medicine and hygiene. 2012;86(3):540–4. 10.4269/ajtmh.2012.11–0283 22403333 PMC3284378

[50] Molaei G, Andreadis TG, Armstrong PM, Anderson JF, Vossbrinck CR. Host feeding patterns of Culex mosquitoes and West Nile virus transmission, northeastern United States. Emerging infectious diseases. 2006;12(3):468–74. 10.3201/eid1205.051004 16704786 PMC3291451

[51] Molaei G, Andreadis TG, Armstrong PM, Bueno R Jr., Dennett JA, Real SV, et al Host feeding pattern of Culex quinquefasciatus (Diptera: Culicidae) and its role in transmission of West Nile virus in Harris County, Texas. The American journal of tropical medicine and hygiene. 2007;77(1):73–81. . 17620633

[52] Kocher TD, Thomas WK, Meyer A, Edwards SV, Paabo S, Villablanca FX, et al Dynamics of mitochondrial DNA evolution in animals: amplification and sequencing with conserved primers. Proceedings of the National Academy of Sciences of the United States of America. 1989;86(16):6196–200. 2762322 10.1073/pnas.86.16.6196 PMC297804

[53] Koella JC, Sorensen FL, Anderson RA. The malaria parasite, Plasmodium falciparum, increases the frequency of multiple feeding of its mosquito vector, Anopheles gambiae. Proceedings Biological sciences / The Royal Society. 1998;265(1398):763–8. 10.1098/rspb.1998.0358 9628035 PMC1689045

[54] Darbro JM, Dhondt AA, Vermeylen FM, Harrington LC. Mycoplasma gallisepticum infection in house finches (Carpodacus mexicanus) affects mosquito blood feeding patterns. The American journal of tropical medicine and hygiene. 2007;77(3):488–94. . 17827365

[55] Gokool S, Smith DF, Curtis CF. The use of PCR to help quantify the protection provided by impregnated bednets. Parasitology today. 1992;8(10):347–50. . 15463534 10.1016/0169-4758(92)90072-a

[56] Gokool S, Curtis CF, Smith DF. Analysis of mosquito bloodmeals by DNA profiling. Medical and veterinary entomology. 1993;7(3):208–15. . 8369554 10.1111/j.1365-2915.1993.tb00678.x

[57] Norris LC, Norris DE. Heterogeneity and changes in inequality of malaria risk after introduction of insecticide-treated bed nets in Macha, Zambia. The American journal of tropical medicine and hygiene. 2013;88(4):710–7. 10.4269/ajtmh.11-0595 23382169 PMC3617857

[58] Soremekun S, Maxwell C, Zuwakuu M, Chen C, Michael E, Curtis C. Measuring the efficacy of insecticide treated bednets: the use of DNA fingerprinting to increase the accuracy of personal protection estimates in Tanzania. Tropical medicine & international health: TM & IH. 2004;9(6):664–72. 10.1111/j.1365-3156.2004.01250.x . 15189456

