# Supplementary figures and images for: Unbiased Characterization of Anopheles Mosquito Blood Meals by Targeted High-Throughput Sequencing
Source: PLoS Negl Trop Dis. 2016 Mar 10;10(3):e0004512. doi: 10.1371/journal.pntd.0004512 (PMC4786206; doi:10.1371/journal.pntd.0004512)

**A**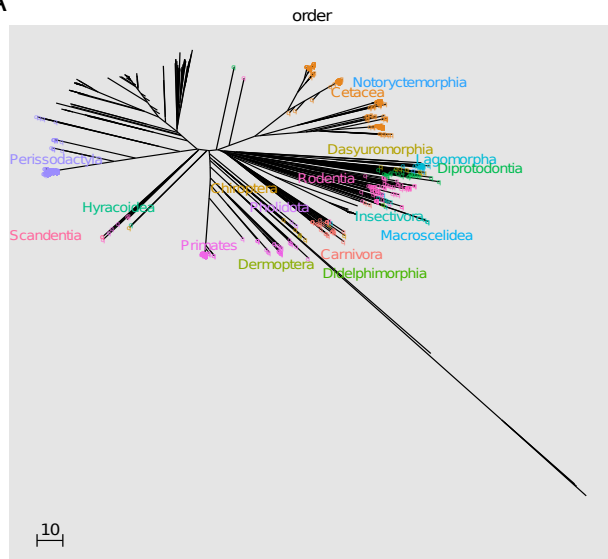**B**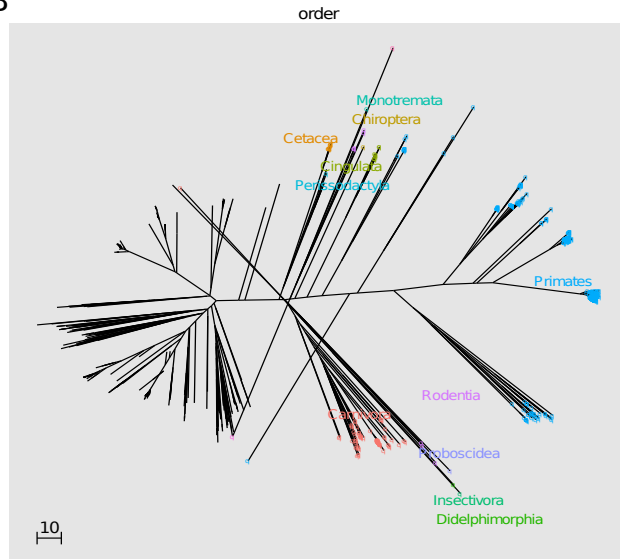**C**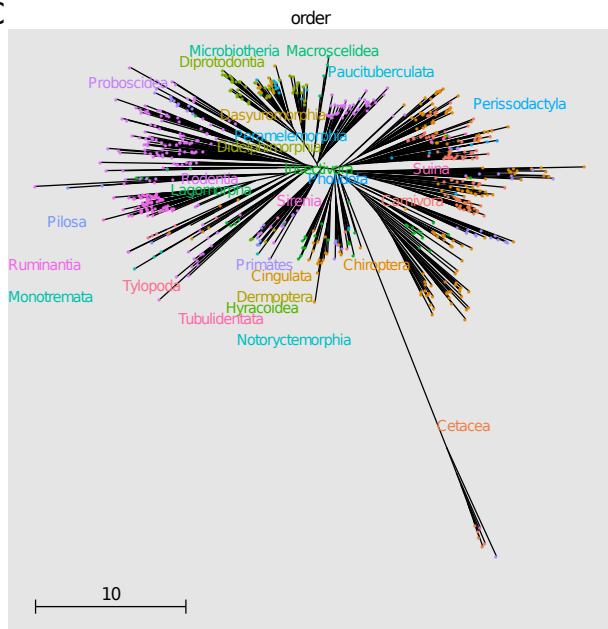**D**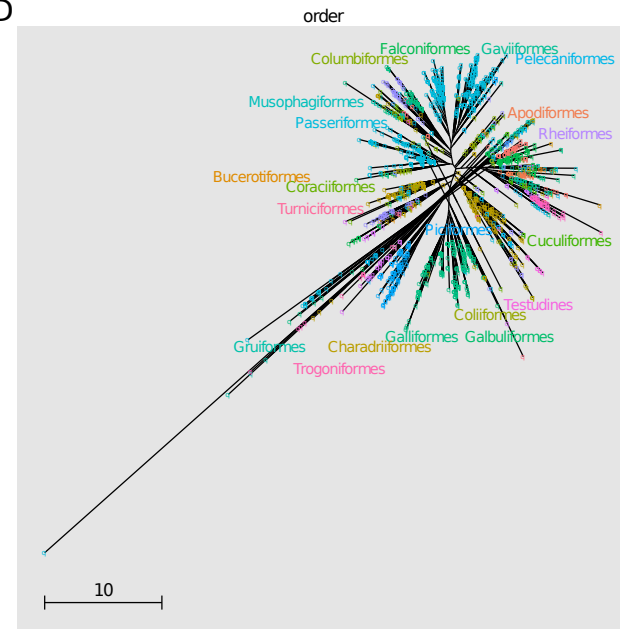

Supplement: S1 Fig — Neighbor-joining tree reconstructed using the DNA sequences predicted to be amplified by primers targeting the mammalian mitochondrial (A) COI, (B) Cytb [37] and (C) 16S rRNA [36] as well as by primers targeting the (D) avian 12S ribosomal RNA [39]. Each colored dot represents a different DNA sequence that is colored according to its taxonomy. Despite being much smaller (140 bp on average vs 704 bp for COI and 819 bp for Cyt B), the 16S rRNA sequences provides similar information content as the other mitochondrial genes. (Note that the short length of the 16S rRNA amplicon greatly facilitates next-generation sequencing). In addition, the number of mammalian species that have been sequenced for 16S rRNA (N = 1,752) is much greater than for the other loci (respectively, 244 and 225 for COI and CytB) enabling more robust species identification. (PDF) [file pntd.0004512.s006.pdf]

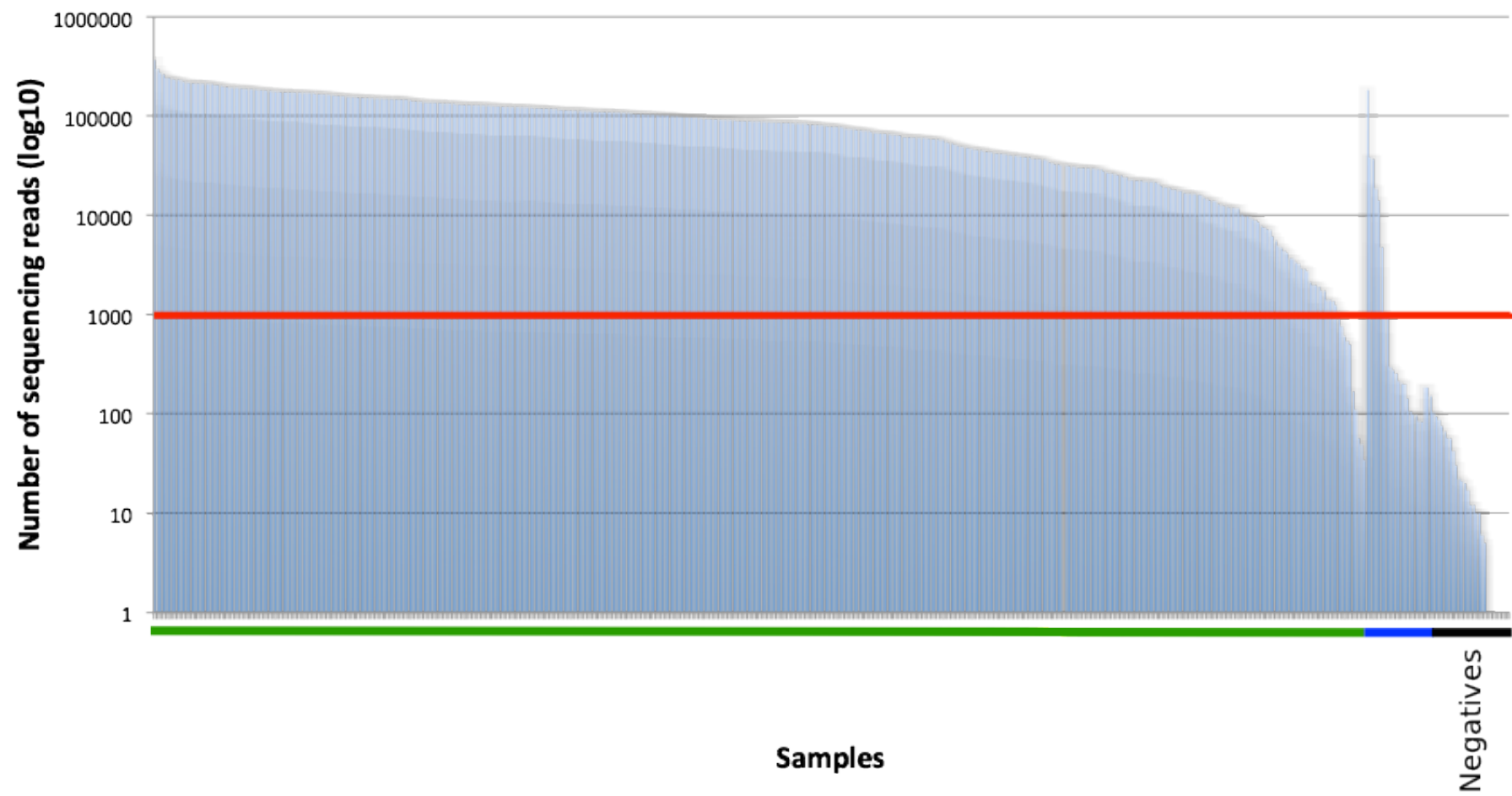

Supplement: S2 Fig — Each vertical bar represents a single mosquito ranked along the x-axis according to the number of reads obtained to characterize its blood meal (y-axis, log scaled). The panel underneath the plot indicates whether the mosquitoes were visually classified as fed (fully-fed and partially-fed, green horizontal bar) or non-fed (blue bar). Extraction controls (water) are represented by the black horizontal bar. The horizontal red bar at 1,000 indicates the cut-off used for analysis inclusion. (PDF) [file pntd.0004512.s007.pdf]

class

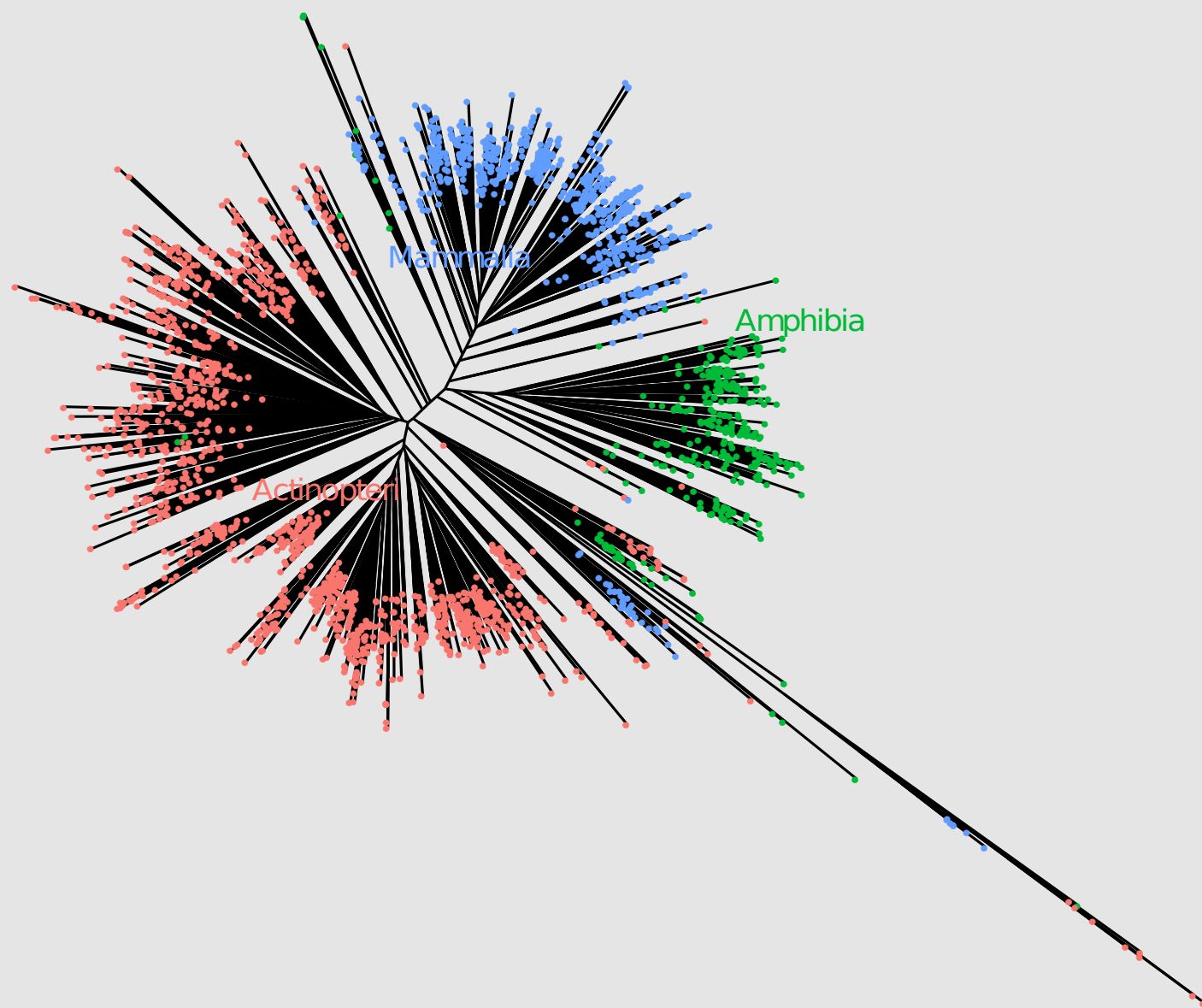

10

Supplement: S3 Fig — Each colored dot represents a different DNA sequence. The tree shows the entire range of species amplified and colored by classes (Blue, mammals; Red, bony-fish; Green, amphibians). (PDF) [file pntd.0004512.s008.pdf]

Number of Sequencing Reads

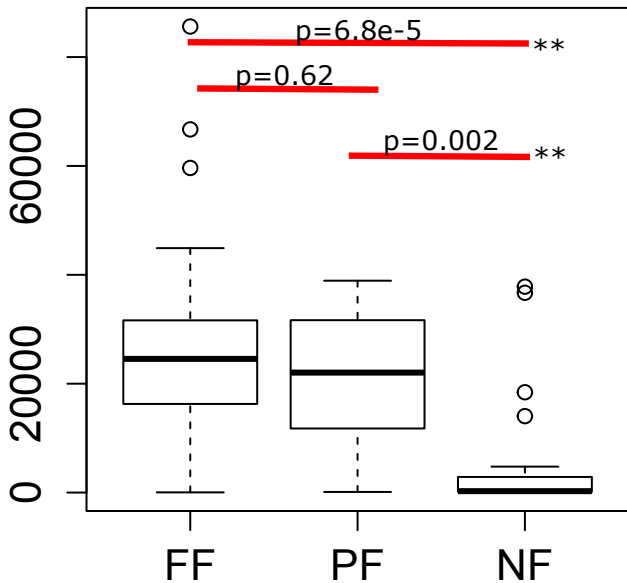

Supplement: S4 Fig — (PDF) [file pntd.0004512.s009.pdf]

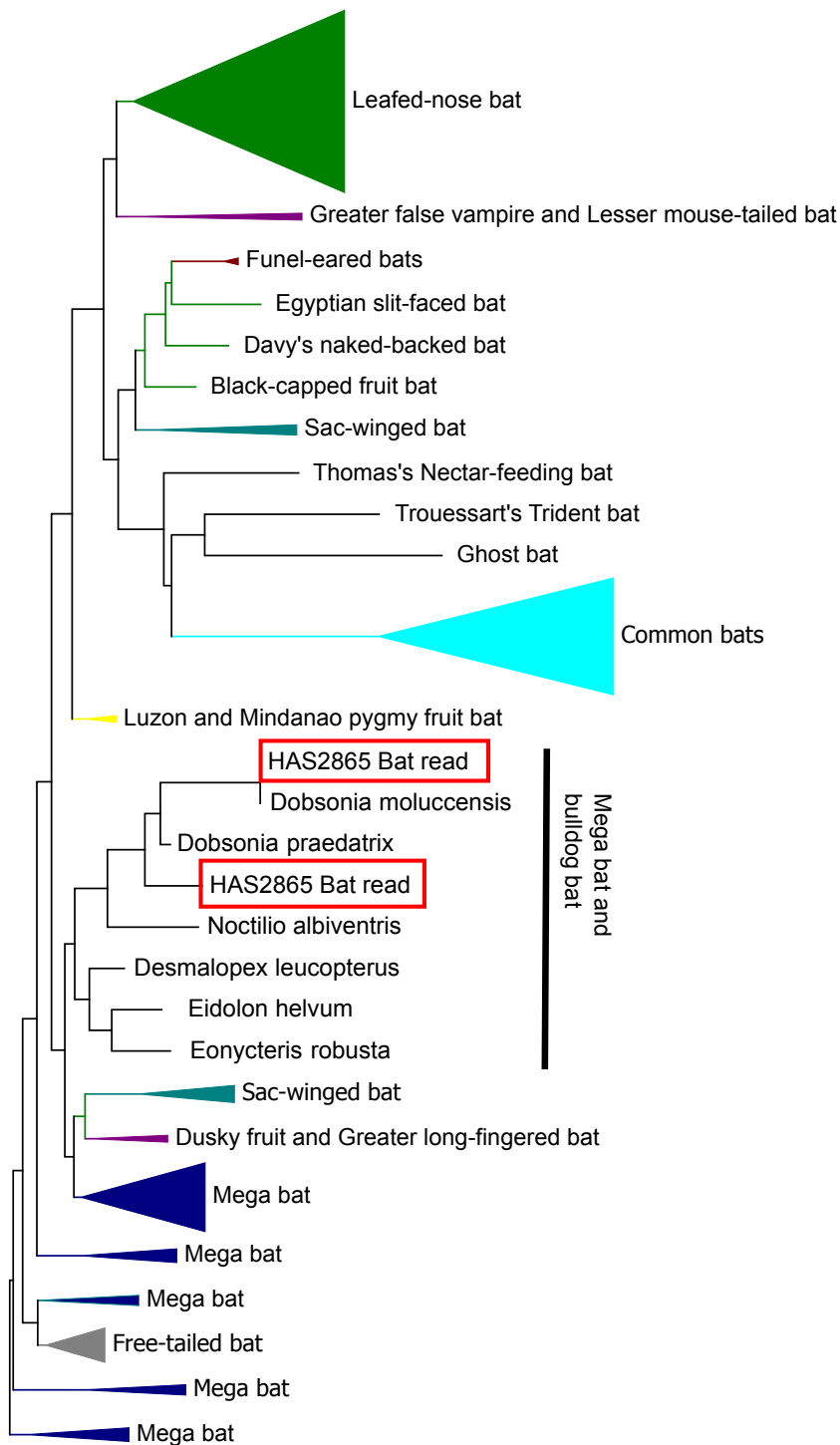

Mega bat and  
bulldog bat

0.05

Supplement: S5 Fig — The two bat DNA sequences amplified from one mosquito’s blood meal are shown by the red boxes. (PDF) [file pntd.0004512.s010.pdf]

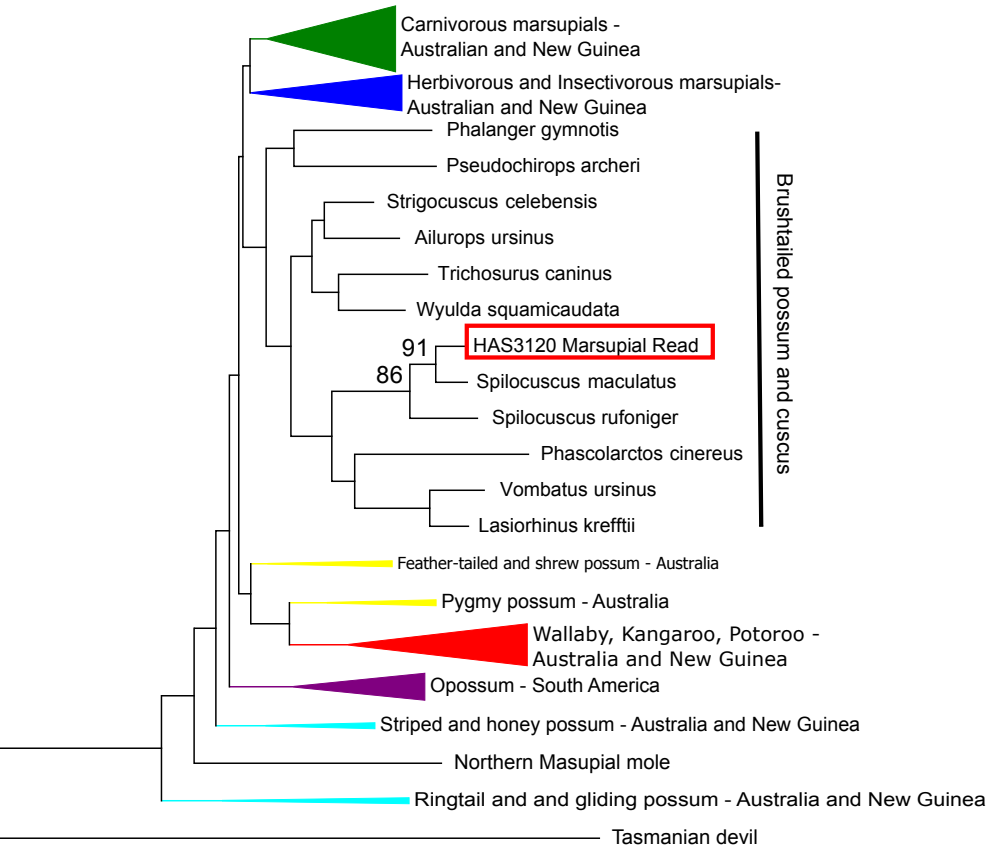

Brush-tailed possum and cuscus

5

Supplement: S6 Fig — The marsupial DNA sequence amplified from one mosquito’s blood meal is shown in the red box. (PDF) [file pntd.0004512.s011.pdf]

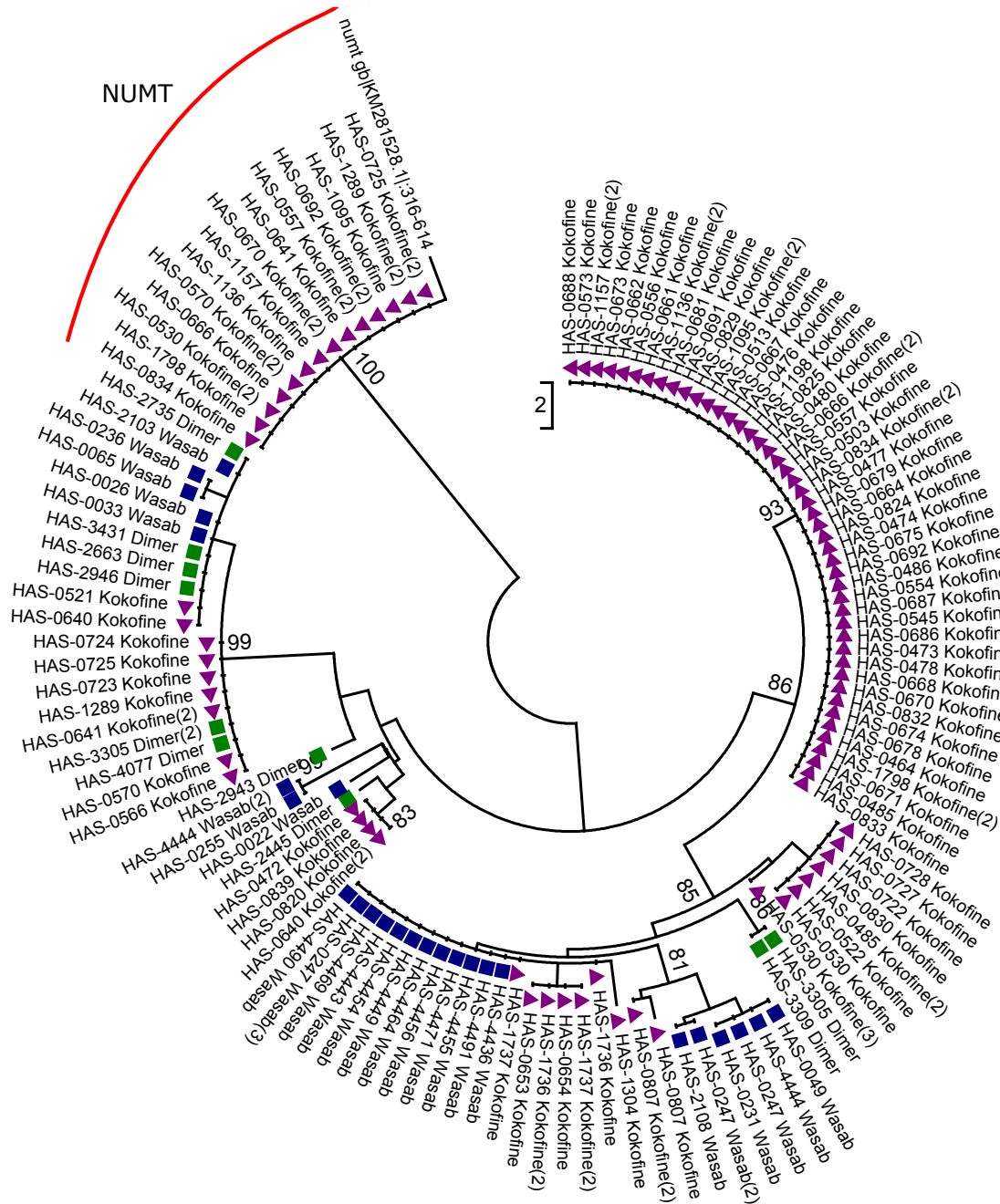

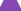 AF4- Kokofine  
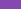 AP- Wasab  
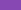 AP - Dimer

Supplement: S7 Fig — The shapes indicate the species of each mosquito carrying a specific human DNA sequence (squares represent An. punctulatus s.s., triangles An. farauti 4). The color of each shape indicates the village where the mosquito was collected (green from Dimer, blue from Wasab, and purple from Kokofine). Note the long-branch separating the mitochondrial DNA sequences from the nuclear insertion (numt) sequence. (PDF) [file pntd.0004512.s012.pdf]
